# Enteric Phageome Alterations in Patients With Type 2 Diabetes

**DOI:** 10.3389/fcimb.2020.575084

**Published:** 2021-01-22

**Authors:** Qian Chen, Xiaojing Ma, Chong Li, Yun Shen, Wei Zhu, Yan Zhang, Xiaokui Guo, Jian Zhou, Chang Liu

**Affiliations:** ^1^ Department of Microbiology and Immunology, Shanghai Jiao Tong University School of Medicine, Shanghai, China; ^2^ Department of Endocrinology and Metabolism, Shanghai Clinical Center for Diabetes, Shanghai Diabetes Institute, Shanghai Jiao Tong University Affiliated Sixth People’s Hospital, Shanghai, China; ^3^ Cancer Institute, Fudan University Shanghai Cancer Center, Department of Oncology, Shanghai Medical College, Fudan University, Shanghai, China; ^4^ School of Global Health, Chinese Center for Tropical Diseases Research, Shanghai Jiao Tong University School of Medicine, Shanghai, China

**Keywords:** bacterial microbiome, lipopolysaccharide, phageome, type 2 diabetes, bacteriophage

## Abstract

Type 2 diabetes is a complex metabolic disease and has been shown to involve alteration of the gut microbiota. Previous studies have primarily focused on changes in the bacterial microbiome, while ignoring the phage community composition. Extracellular phages can lyse host bacteria and thus influence the microbiota through positive or negative interactions with bacteria. We investigated changes in the extracellular phageome and discussed its role in T2D pathogenesis. We used a sequencing-based approach to identify bacteriophage after isolation of VLPs (virus like particles) from fecal samples. We identified 330 species of phages according to the predicted host bacteria from T2D patients (N=17) and nondiabetic controls (N=29). The phageome characteristics were highly diverse among individuals. In the T2D group, the intestinal phage population was altered, and the abundance of phages specific to *Enterobacteriaceae* hosts increased markedly. Meanwhile, the abundance of *Enterobacteriaceae* in the gut was significantly increased, and systemic LPS content elevation was observed in the T2D group. Additionally, a consortia of eight phages was found to distinguish T2D patients from nondiabetic controls with good performance (AUC>0.99).

## Introduction

Type 2 diabetes (T2D) has been one of the leading health issues globally in recent decades (Defronzo et al., 2015). It is characterized by hyperglycemia in the context of insulin resistance and impaired insulin secretion, which results from a complex inheritance-environment interaction along with other risk factors, such as sedentary behavior, obesity, and unhealthy dietary habits. T2D and its complications affect almost all populations in both developed and developing countries, with high rates of diabetes-related morbidity and mortality (Zheng et al., 2018). In the global morbidity data in 2015, the number of adult (20–79 years) patients with diabetes mellitus was the highest in China (109.6 million), representing 10.6% of the domestic population, followed by India and the USA (Ma et al., 2014; Foos et al., 2019).

As highlighted by many previous studies, the gut microbiome has been proven to play a fundamental role in metabolic disorders, including T2D (Barlow et al., 2015; Arora and Backhed, 2016; Sharma and Tripathi, 2019) and obesity (Turnbaugh et al., 2006; Turnbaugh et al., 2009; Dao and Clément, 2018; Thaiss, 2018; Cani, 2019), and even cardiovascular diseases (Brown and Hazen, 2018; Cosola et al., 2018; Joris and Gloor, 2019). Shifts in the gut microbiome can result in increased gut permeability, altered metabolism of short-chain fatty acids (SCFAs) and vitamins, and dysfunction in glucose modulation, lipid homeostasis, satiety regulation, and energy production (Pascale et al., 2018; Sharma and Tripathi, 2019). For example, low vitamin D production in the gut has been associated with an increased risk of T2D (Lu et al., 2009; Martini et al., 2010; Lim et al., 2013), and butyrate produced by the gut microbiota is inversely associated with the degree of insulin resistance (Gao et al., 2009; Lin et al., 2012). Moreover, acetate has been proven to regulate the functions of islet β-cells by mediating a feedback loop related to metabolic syndrome in a microbiota-dependent way (Perry et al., 2016). Evidence from both human studies and animal trials has illuminated that T2D is associated with lipopolysaccharide (LPS) released from gram-negative bacteria translocating across the impaired gut barrier with hyperpermeability caused by inflammation, which results in a moderate increase in serum LPS content (Gubern et al., 2006; Cani et al., 2008; Alattas et al., 2009). LPS induces chronic low-grade inflammation and is associated with leptin and insulin resistance, which contributes to the establishment of T2D (Zhou et al., 2018).

It is well known that bacteriophages, the dominant constituent of the virome, inhabit with a high abundance in the intestinal microbiota, with phage-bacterium ratios of ~1:1 (Carding et al., 2017). The biological characteristics of phages endow them with the capability to regulate the abundance of their hosts, thereby affecting the structure of the microbiota through the cascade reactions of both positive and negative interactions among the bacterial communities. Additionally, phages can influence intestinal metabolome features through the altered bacterial communities (Hsu et al., 2019). Although bacteriophages are viruses that infect bacteria, in addition to affecting human health *via* manipulating bacterial community, they have been proved to penetrate epithelial cell layers, aggravate intestinal inflammation, and play a potential role as new mammalian pathogen to affect human health and disease directly (Nguyen et al., 2017; Tetz et al., 2017; Gogokhia et al., 2019). On the other hand, gram-negative phage hosts can result in an increase in LPS content (Tetz and Tetz 2018). The phage community deserves additional attention in research on T2D, for its contribution in regulating both the microbiota and circulating LPS content, as well as the potential pathogenic roles (Gogokhia et al., 2019).

**Figure 1:**
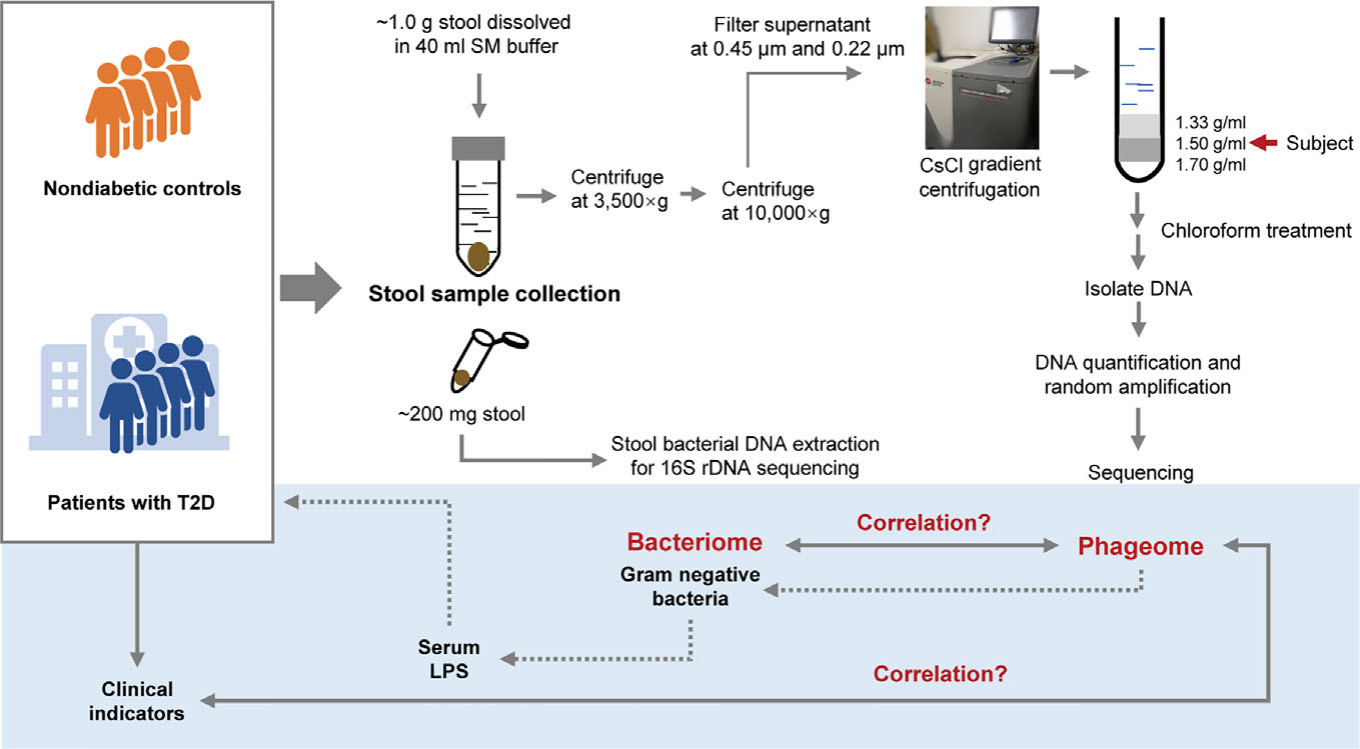
Schematic diagram of this study. The light-blue part is a sketch map of the preliminary exploration herein, including exploring the relationship between the phageome and putative host bacterial microbiome, correlation between the phageome and type 2 diabetes (T2D) disease indicators, and discussion of the potential connection between the increase in serum lipopolysaccharide (LPS) content and the changes in bacteriophages.

Since extracellular phages can infect and lyse bacterial hosts and directly influence the composition of the microbiota, we adopted the VLP isolation-dependent method and attempted to explore the changes in the gut extracellular phage community in a cohort with T2D in Shanghai, China. In addition, whether the changes in the phageome are assciated with the changes in bacterial community was investigated and then the potential relationship between the alteration of the phageome and the increased serum LPS content was discussed. A study design diagram is shown in **Figure 1**.

## Materials and Methods

### Ethics Statement

The study was approved by the Ethics Committees of Shanghai Jiao Tong University Affiliated Sixth People’s Hospital. This study was registered at www.chictr.org.cn with clinical trial registration number ChiCTR-IPR-17011324. All subjects provided written informed consent before enrollment.

### Human Subjects

Patients with T2D involved in this study were recruited from Shanghai Jiao Tong University Affiliated Sixth People’s Hospital. Fecal samples and serum samples were collected, divided into two aliquots and stored in a −80°C freezer. Healthy volunteers aged 20 to 50 years old in the control group were recruited from Shanghai Jiao Tong University Affiliated Sixth People’s Hospital. All subjects in the nondiabetic control group were confirmed without diabetes by oral glucose tolerance test (OGTT) and excluded for other metabolic diseases. The mean fasting plasma glucose, 2 h plasma glucose and hemoglobin A1c levels were 5.05 ± 0.36 mmol/L, 5.90 ± 1.00 mmol/L and 5.2 ± 0.2% respectively. Those who received antibiotics in the preceding 3 months were excluded from this study. The clinical characteristics of the T2D patients are presented in **Table 1**. The detailed test results of nondiabetic controls are not shown in this study, while the relevant clinical features, medications and demographic information recorded for each patient are listed in **Supplementary Table 6**.

**Table 1:** Clinical characteristics of the type 2 diabetes (T2D) patients involved in this study.

Variables	Values in T2D (mean ± SD)
Blood pressure (mmHg)	
Systolic	132 ± 21
Diastolic	77 ± 6
Plasma glucose (mmol/L)	
Fasting	7.24 ± 2.34
0.5 h post meal	10.23 ± 1.90
2 h post meal	12.41 ± 4.22
Insulin (μU/ml)	
Fasting	28.98 ± 45.03
0.5 h post meal	78.01 ± 80.18
2 h Post meal	98.02 ± 88.61
Hemoglobin A1c (%)	8 ± 2
Serum triglycerides (mmol/L)	1.45 ± 0.75
Serum cholesterol (mmol/L)	
Total	4.32 ± 1.09
LDL-c	2.65 ± 0.97
HDL -c	1.07 ± 0.22
Free thyroxine (pmol/L)	16.87 ± 2.61
Hypersensitive C-reaction protein (mg/L)	0.90 ± 1.20

### Diagnosis of Diabetes

We used the 2010 American Diabetes Association (ADA)’s criteria American Diabetes Association (2010) for the diagnosis of diabetes. Diabetes was defined as fasting glucose ≥ 7.0 mmol/L and/or 2-h glucose ≥ 11.1 mmol/L and/or HbA1c ≥ 6.5%. Those using antidiabetic drugs upon examination were also included in the T2D group.

### Sample Treatment for VLP Isolation and DNA Extraction

One aliquot of the stool samples was weighed to approximately 1.0 g and placed in sterile SM buffer (100 mM NaCl, 8 mM MgSO_4_•7H_2_O, 50 mM Tris-Cl (pH 7.5), 0.01% gelatin (w/v)). Then, the fecal samples were vortexed until they were thoroughly mixed with SM buffer. The sample treatment process was based on previous studies (Thurber et al., 2009; Reyes et al., 2010; Minot et al., 2011). In brief, the sample homogenates were centrifuged (3,500×g for 30 min at 4°C). Following another centrifugation (10,000×g for 20 min at 4°C) of the supernatants, the resulting supernatants were collected and filtered through a 0.45 μm and 0.22 μm Millipore filter sequentially. CsCl was added to the filtrate to a final density of 1.15 g/ml. Then the filtrate was deposited on top of a 3 ml discontinuous CsCl gradient, which was prepared using 1 ml CsCl solutions with densities of 1.70 g/ml, 1.50 g/ml, and 1.35 g/ml in SM buffer respectively. CsCl solutions were deposited in the centrifuge tube from the bottom to the top according to the density. The samples were centrifuged (33,000 rpm for 4 h at 4°C) in a SW41Ti swinging bucket rotor (Beckman). The 1.50 g/ml layer was recovered for enriched bacteriophages (Thurber et al., 2009). This procedure is illustrated in **Figure 1**.

The 1.50 g/ml layer was collected from the step gradient and used for the source of the phageome. Using a method with phenol/chloroform/isoamyl alcohol described previously (Reyes et al., 2010), the DNA was extracted from each sample. The extraction method can be described as follows. Chloroform (0.2 volumes) was added to the 1.50 g/ml layer collected from the step gradient. The aqueous phase was collected after centrifugation (5 min at 3,000×g), and treated with DNase. Subsequently, 2M Tris HCl/0.2M EDTA (0.1 volumes), formamide (1 volume) and 100 ml of a 0.5M EDTA solution were added per 10 ml of sample, and the resulting mixture was incubated at room temperature for 30 min. Ethanol (2 volumes) was added to wash the sample, which was then pelleted by centrifugation (15 min at 10,000×g at 4°C). The precipitate was washed with 70% ethanol and resuspended in 567 μl TE buffer, and then 30 μl of 10% SDS solution and 3 μl of proteinase K (20 mg/ml) were added thereto. After an incubation for 1 h at 55°C, 100 μl of 5M NaCl and 80 μl of 10% cetyltrimethylammonium bromide/0.7M NaCl were subsequently introduced to the mixture, and incubate for a 10 min at 65°C. The mixture was centrifuged (5 min at 8,000×g) after an equal volume of chloroform was added. The supernatant was recovered and an equal volume of phenol:chloroform:isoamyl alcohol (25:24:1, v:v) was added, followed by another centrifugation (5 min at 8,000×g). An equal volume of chloroform was introduced to the recovered supernatant, and the mixture was centrifugated. Then the supernatant was collected, and 0.7 volumes of isopropanol was added for precipitating the DNA. DNA was pelleted after centrifugation (15 min at 13,400g at 4°C), and was washed with cold 70% ethanol, air-dried, and resuspended in 30 μl TE buffer.

Before DNA sequencing, the total DNA was amplified using a GenomiPhi V2 kit (GE Healthcare) to increase the DNA level.

### *16S rRNA* Gene Sequencing

Total DNA from one aliquot of the fecal samples was extracted using a QIAamp Fast DNA Stool Mini Kit followed the operation handbook. Then, *16S rRNA* high-throughput sequencing was performed using an Illumina HiSeq PE250. The variable regions V3–V4 on *16S rRNA* genes in bacterial genomes were amplified with the forward primer F341 5’-ACTCCTACGGGRSGCAGCAG-3’ and reverse primer R806 5’-GGACTACVVGGGTATCTAATC-3’. The raw paired end reads were assembled by pandaseq with overlapping nucleotides. Then, the reads were quality filtered. The raw data were then subjected to a quality control procedure using UPARSE. The qualified reads were clustered to generate operational taxonomic units (OTUs) at the 97% similarity level using Usearch in Qiime1 pipeline (Caporaso et al., 2010). RDP (Ribosomal Database Project) was used for bacterial taxonomic classification (Cole et al., 2014). At the same time, the chimera filtering was performed. Principal component analysis (PCA), heatmap analysis, Bray-Curtis similarity clustering, and species abundance analysis were performed using the R program.

### Metagenomic Sequencing of DNA From Phages Derived From VLPs

Subject phage DNA was first sheared into ~400 bp-long fragments with a Covaris S2 instrument (Covaris, US). The resulting DNA fragments were used to construct a sequencing library according to the manufacturer’s instructions (NEXTflex™ DNA Sequencing Kit compatible with the Biomek^®^ FXp (Bio Scientific, US)). DNA libraries were sequenced on an Illumina^®^ X-ten platform with a read length of 150 bp.

### Assembly of VLP Contigs and Their Quantitation at Various Taxonomic Levels

Raw reads for each sample were preprocessed using *Trimmomatic* software (Bolger et al., 2014) to trim adapters and remove reads with low quality and insufficient length. Given a large read data from metagenomic sequencing, we used two-step assembly method. In detail, the resulting (clean) reads above were first assembled into pre-contigs for each sample using velvet software (version, 1.2.10) (Zerbino and Birney, 2008). BLASTx in the Blast+ package (Camacho et al., 2009) was then performed to find hits of these pre-contigs in a database of phage orthologous groups (POGs) with an e-value cut-off of 10 (Kristensen et al., 2013). The POGs includes a BLAST-formatted searchable database which incorporate genomes of double-stranded DNA (dsDNA) phages, single-stranded DNA (ssDNA) and ss- and dsRNA phages, as well as archaeal viruses and could recognize taxon-specific signatures for virus classification with specificity (Kristensen et al., 2013). The clean reads from each sample were mapped to the pre-contigs which were hit in the POGs with bowtie2 (Langmead and Salzberg, 2012), and the mapped reads were retained. To obtain unified contigs for comparison with samples, the mapped reads from each sample were pooled to re-assembly using velvet software. The resulting contigs with length more than 200 base pairs (bps) were kept. Finally, taxonomic information including order, family, genus, and species for VLP contigs was obtained. Once one VLP contig was hit in POGs database *via* BLASTx, taxonomic information would be assigned and host bacterium of this VLP contig was directly supplied at phage species level.

The clean reads for each sample above were separately re-aligned to the VLP contigs using bowtie2 (Zerbino and Birney, 2008) and the abundance (counts of mapped reads) of the VLP contigs of each sample was generated by samtools software (Li et al., 2009), resulting viral abundance tables at various taxonomic levels. The same types (ds DNA, ssDNA, or unclassified phages) were collapsed, and the order, family, genus and species with the same annotated name at different taxonomic levels were simply merged. Correspondingly, abundance (read counts) of the type or taxonomic levels was summed and normalized abundances of them were obtained using reads of VLP contigs per million mapped reads (RPM).

### Calculation of α- and β-Diversity of the Phage Communities

The α-diversity was calculated based on the VLP abundance data using summary.single command line in mothur software (Schloss et al., 2009), resulting in α-diversity index including Ace, Chao, Shannon and Simpson. β**-**diversity was obtained by dist.shared command based on thetaYC distances in mothur software.

### Circulating LPS Level Measurement

Serum LPS was assayed using a chromogenic TAL test, which is a quantitative test for gram-negative bacterial LPS (BIOENDO, Xiamen, China). Gram-negative bacterial LPS catalyzes the activation of a proenzyme in the LAL. The activated enzyme catalyzes the splitting of p-nitroaniline (pNA) from the colorless substrate Ac-Ile-Glu-Ala-Arg-pNA. The pNA released was measured photometrically at 405-410 nm following termination of the reaction. The correlation between the absorbance and the LPS concentration was linear in the 0.1-1.0 EU/ml range. All samples were assessed in duplicate within the same plate.

### Serum TNF-α and IL-6 Measurement

Serum TNF-α and IL-6 concentrations were measured with human TNF-α ELISA kit (R&D systems, Minneapolis, USA) and IL-6 ELISA kit (R&D systems, Minneapolis, USA), respectively. For detailed protocol, please refer to handbooks of the products.

### Statistical Analysis

Statistical analyses were performed using R software and GraphPad Prism. Random forest models were generated with the AUCRF package (Calle et al., 2011). T-tests were performed to assess differences in the α-diversity of the T2D group and Ctrl group (Rosa et al., 2015; Xia et al., 2018). For comparison of continuous variables, the Mann-Whitney U-test was conducted for two groups. Multiple test correction was performed using the Benjamini and Hochberg method (Benjamini and Hochberg, 1995), and the false discovery rate (FDR) was obtained. Spearman’s rank test was performed for correlation analysis. Pearson correlation analysis was used to evaluate the correlation between bacteriophages and the corresponding bacterial hosts (Hazra and Gogtay, 2016).

## Results

### Fecal Phageome Composition Based on Metagenome Sequencing

A total of 46 samples, including those from 17 T2D and 29 control subjects, were collected. On average, 11.7 Gb ( ± 2.14 Gb, SD) were sequenced for each sample. From these sequences, we obtained 3,136 phage contigs with an average length of 7,257 bp, resulting in a total of 330 defined phage species (**Supplementary Table 1**). In total, 89.5% of the detected genomes of the phages were in the form of double-stranded DNA (dsDNA), and less than 0.3% existed as single-stranded DNA (ssDNA). The fecal phage composition was highly variable between individuals in the T2D (D) group and nondiabetic controls (Ctrl) group. There was a higher percentage of ssDNA in D17 sample (24.3%) than in the remaining samples (**Supplementary Figure 1A**). *Caudovirales* accounted for the most abundant phage community at the order level (89.8% on average). Especially in D03 sample, almost all of the phages detected were defined as *Caudovirales* (>99.9%). In H18 sample, *Caudovirales* composed only 64.1% of the phage population (**Supplementary Figure 1B**).

At the family level, five phage families, *Myoviridae*, *Podoviridae*, *Microviridae*, *Siphoviridae*, and *Tectiviridae*, were detected. In most samples, the percentage of *Siphoviridae* was the highest, with an average of 58.7%, followed by *Myoviridae* (25.7%). D03 sample and D15 sample contained almost only the families *Podoviridae* and *Siphoviridae*, respectively. Moreover, the abundance of *Microviridae* detected in D17 sample and D08 sample was higher than that in other samples (**Supplementary Figure 1C**).

Furthermore, these detected viral sequences were aligned to 27 viral genera. Similar to the above, at the genus level, the compositions of the virome in the samples were highly diverse. Certain viral genera were dominant in several samples, resulting in low diversity indexes. The relative abundances of T1-like virus in D02 sample, T4-like virus in D17 sample, H18 sample, and H24 sample, phiKMV-like in H12 sample, and Lambda-like viruses in D07 sample and D09 sample were particularly high (**Supplementary Figure 1D**). Detailed information is shown in **Supplementary Table 2**.

### Phageome Characteristics in T2D Patients

**Figure 2:**
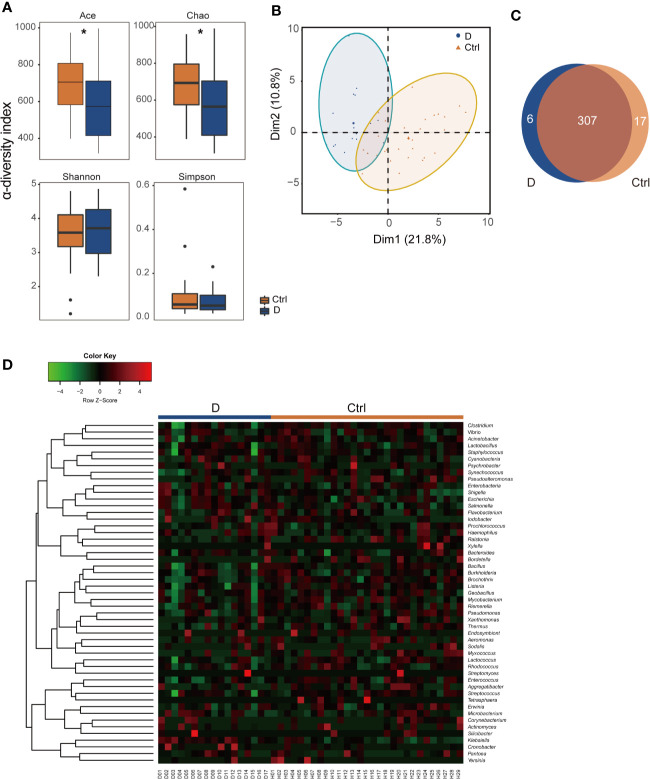
Illustration of the identified phageome characteristics. **(A)** α-diversity indexes of the phageome in the type 2 diabetes (T2D) group (N=17) and Ctrl group (N=29). **(B)** Principal component analysis (PCA) of the phageome in the T2D group and Ctrl group. PCA plot of phageome samples from diabetes patients (blue dots) and controls (orange triangles). The first component (dim1) explained 21.8% of the total variance, and the dim2 explained 10.8%. **(C)** Venn diagram for the number distribution of phages classified by bacterial hosts in the two groups. **(D)** Heatmap of the identified phages in T2D patients and nondiabetic controls according to their corresponding bacterial hosts. *p < 0.05.

α-Diversity was assessed with 4 different diversity analysis methods. Both the Ace and Chao1 index were significantly reduced in the T2D group, while the Shannon and Simpson index showed no obvious changes between the two groups (**Figure 2A**). Regarding the results of β-diversity analysis, significantly different phageome characteristics were observed between T2D patients and nondiabetic populations based on principal components analysis (**Figure 2B**).

The phages detected were classified according to their putative bacterial hosts. We defined a total of 330 species of phage with 51 putative bacterial hosts, among which there were 313 in the T2D group and 324 in the Ctrl group, with 307 species found in both groups (**Figure 2C**). The abundances of these phages in the T2D and Ctrl groups were clustered to obtain a heatmap, as shown in **Figure 2D**.

### Alterations in Phage Communities in Type 2 Diabetes Patients

**Figure 3:**
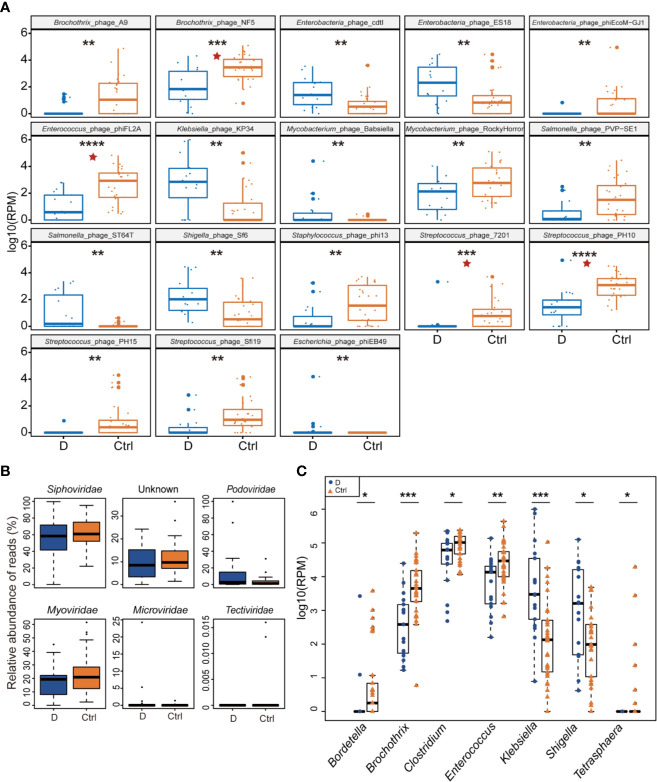
Differential abundance of phage-infected bacterial taxonomy between type 2 diabetes (T2D) patients (N=17) and controls (N=29). **(A)** The 18 bacteriophages with the most significant difference in the T2D group. **(B)** Differentially detected viruses at the level of families. **(C)** Phages clustered by 7 bacterial genus hosts that changed significantly in the T2D group. *p< 0.05, **p< 0.01, ***p< 0.001, ****p< 0.001. pentagram: FDR < 0.05.

Of the 330 species of bacteriophage identified sorting by their bacterial hosts (**Supplementary Table 1A**), the abundance of 58 species of phage was significantly different between T2D patients and nondiabetic controls (**Supplementary Table 1B**). There were 52 species with a cutoff of an FDR<0.25 and four bacteriophages, *Brochothrix*_phage_NF5, *Enterococcus*_phage_phiFL2A, *Streptococcus*_phage_PH10, and *Streptococcus*_phage_7201, with an FDR<0.05 between the two groups (**Supplementary Table 1B**). The 18 phages with the most significant differences are shown in **Figure 3A**.

After being clustered according to the level of the family, shifts in abundances of phage families in the T2D group versus the Ctrl group were compared, and no statistically significant differences were observed in dsDNA phages including *Myoviridae*, *Podoviridae*, *Siphoviridae*, and *Tectiviridae*. Meanwhile, there’s no significant difference in abundance of *Microviridae*, the most abundant ssDNA phages detected in this study (**Figure 3B**).

Host bacterial assignments for curated phage contigs were compared at the genus level between the two groups; seven host bacterial genera were significantly changed (p ≤ 0.05), namely, *Brochothrix, Klebsiella, Enterococcus, Bordetella, Shigella, Clostridium*, and *Tetrasphaera* (**Figure 3C**). The phages infecting *Brochothrix* and *Klebsiella* had an FDR <0.05 between the T2D group and the Ctrl group.

### Fecal Bacterial Microbiome Features and Their Alterations in T2D Patients

**Figure 4:**
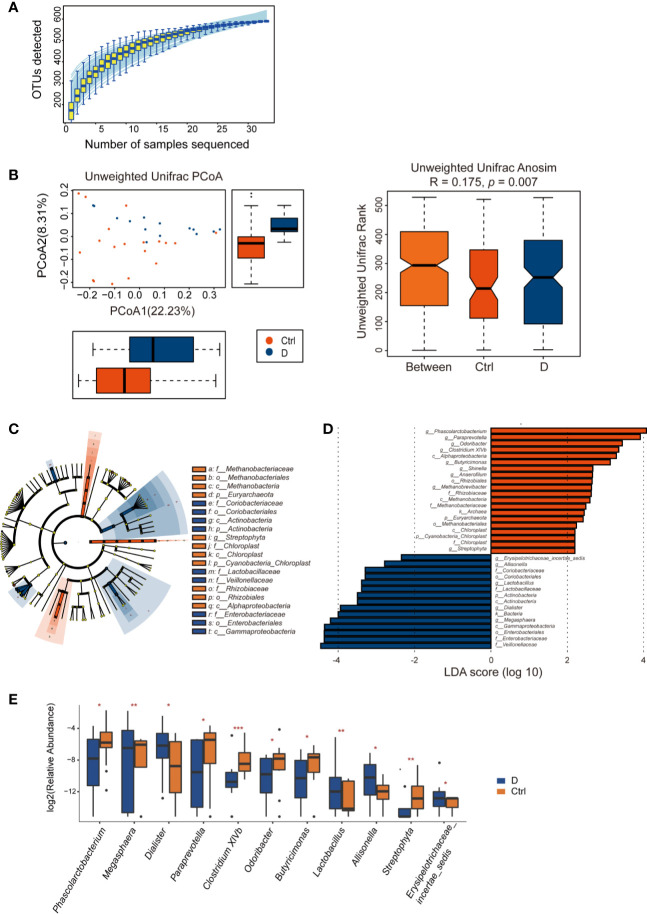
Alterations in the fecal bacterial microbiome in the study. **(A)** Species accumulation curve for the fecal samples. **(B)** Unweighted UniFrac principal coordinate analysis (PCoA) and analysis of similarities (ANOSIM) of the bacterial microbiome in the type 2 diabetes (T2D) group (N=17) and Ctrl group (N=20) taxonomic cladogram **(C)** and LDA **(D)** were obtained using LEfSe of the 16S sequences in the type 2 diabetes (T2D) group (in blue) and Ctrl group (in orange). **(E)** Significantly altered bacterial genera in the T2D group. *p < 0.05, **p < 0.01, ***p < 0.001.

Length of the 16S rRNA amplicon is 400–440 bp. Clean reads were aligned to OTU sequences, which were then counted in each sample. 590 OTUs were mapped in the 33 samples. Among those OTUs, 539 OTUs were detected in Ctrl group, while 413 OTUs in T2D group. Species accumulation curve reached a plateau for bacterial species, indicating that the sample size was sufficient to reveal the bacterial population (**Figure 4A**). The rarefaction curve for each sample based on Chao1 index were shown in **Supplementary Figure 2**. The α diversity of the T2D group showed no significant changes (**Supplementary Figure 3**). Unweighted UniFrac analysis of similarities (ANOSIM) displayed a significant difference in β-diversity between the two groups. Unweighted UniFrac principal coordinate analysis (PCoA) (left) and ANOSIM (right) illustrated that the cluster of fecal bacteria in the T2D group was clearly distinct from that in the nondiabetic control group (**Figure 4B**). The bacteria identified in this study were from 11 phyla. The detailed information is shown in **Supplementary Table 3**.

LEfSe was used for identifying the most varied abundant bacterial taxa at different levels and for analyzing data and visualizing key species that were identified as differentiating factors between diabetes patients and health controls (**Figure 4C**). With a log LDA score above 2.0, we found an enriched abundance of OTUs contributed by *Phascolarctobacterium*, *Paraprevotella*, *Odoribacter*, *Clostridium XIVb*, *Butyricimonas*, *Shinella*, *Anaerofilum*, *Methanobrevibacter*, and *Streptophyta* among health controls, while the T2D patients had increased abundances of *Erysipelotrichaceae_incetae_sedis*, *Allisonella*, *Lactobacillus*, *Dialister*, and *Megasphaera*. Moreover, Members of *Enterobacteriaceae* were enriched in the T2D group (**Figure 4D**).

A total of 129 bacterial genera were identified by *16S rRNA* sequencing. Due to the technological limitations of *16S rRNA* sequencing, among the 129 bacterial genera, 24 phylogenetic types of bacteria failed to be identified to the exact genera. Among the 105 genera, the abundance of 14 genera was significantly different between the two groups. Excluding *Methanobrevibacter*, *Anaerofilum*, and *Shinella*, which could not be detected in the T2D group, we calculated the differences in abundance of the other 11 bacterial genera between the T2D group and Ctrl group. The results are presented in the boxplot in **Figure 4E**.

### Correlations Between Type 2 Diabetes-Associated Changes in the Phageome and Bacterial Microbiome

In this study, the relationship between the phageome and the bacterial microbiome was also investigated. Using *16S rRNA* sequencing, 105 bacterial genera were identified. At the same time, the phageome we identified corresponded to 51 bacterial genus hosts.

**Figure 5:**
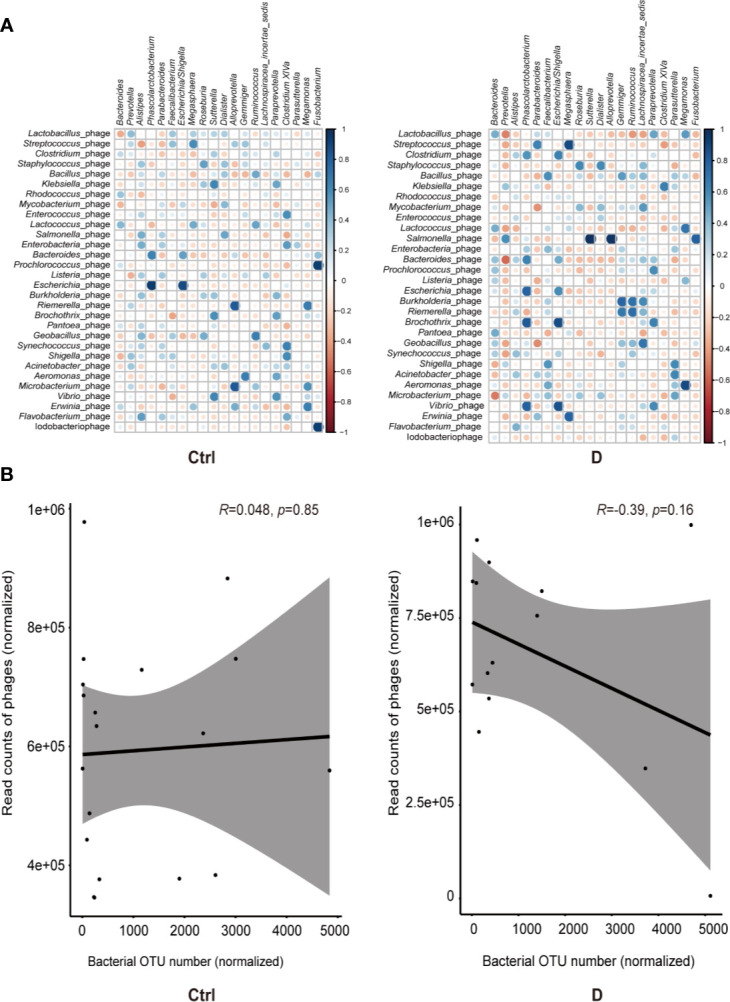
Bacterium-phage correlations in fecal samples from type 2 diabetes (T2D) patients and nondiabetic controls. **(A)** Results of Spearman correlation analysis between the most abundant 20 bacterial genera and the most abundant 30 species of phage. Blue circles indicate positive correlations, and red circles indicate negative correlations. The size and shading indicate the magnitude of the correlation, where darker shades denote more intensive correlations than light ones. **(B)** Correlations of the relative abundance of phages and their bacterial hosts in the T2D group and Ctrl group. The relative abundance was represented by the normalized operational taxonomic units (OTU) number. Optimal regression lines for the 95% confidence interval are plotted.

Spearman correlation analysis was used to assess the correlations between the most abundant bacterial genera and bacteriophages in both the T2D and control groups. Moderately increased correlations in bacteria and phages were found in the T2D group (**Figure 5A**).

The bacteria detected and the hosts of bacteriophages did not completely overlap, and only 16 putative host genera were identified in the detected bacterial group. These bacterial genera were *Klebsiella*, *Enterococcus*, *Lactococcus*, *Aggregatibacter*, *Pseudomonas*, *Pseudoalteromonas*, *Rhodococcus*, *Bacteroides*, *Bacillus*, *Corynebacterium*, *Staphylococcus*, *Streptococcus*, *Lactobacillus*, *Acinetobacter*, *Actinomyces*, and *Haemophilus.*

Pearson correlation analysis was used to evaluate the possible linear relation between bacteriophages and their host bacteria. The results indicated no significant linear correlation between them (**Figure 5B**).

### Contents of Circulating LPS and Pro-Inflammation Cytokines

**Figure 6:**
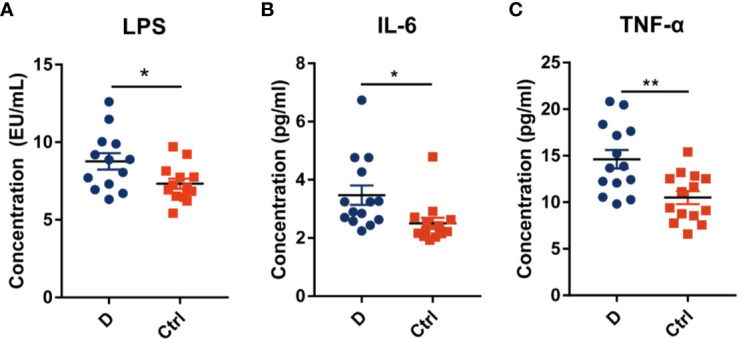
Circulating endotoxin and pro-inflammation cytokines levels in serum. **(A)** The concentration of circulating endotoxin. In each group, N=14. Quantitative analysis of serum IL-6 **(B)** and TNF-α **(C)** in type 2 diabetes patients and nondiabetic controls. N=14 for each group. All data are expressed as mean ± SEM. *p < 0.05, **p < 0.01.

The LPS concentration in serum samples from T2D patients (N=14) and nondiabetic subjects (N=14) was assessed. The results showed a significant increase in the serum LPS concentration in T2D patients (**Figure 6A**), as demonstrated in many studies (Liu et al., 2014; Gomes et al., 2017). Additionally, serum IL-6 and TNF-α levels were significantly increased in T2D subjects (**Figures 6B, C**). We hypothesized that the elevated level of serum LPS in T2D patients may partially result from the intensified lysis of intestinal gram-negative bacteria under the action of phages. Based on this hypothesis, we analyzed the difference in the abundance of the phageomes mapped to gram-negative bacterial phages and gram-positive bacterial phages (**Supplementary Table 1C**).

### The Alteration in Gram-Negative Bacteria and *Enterobacteriaceae*-Specific Phages

**Figure 7:**
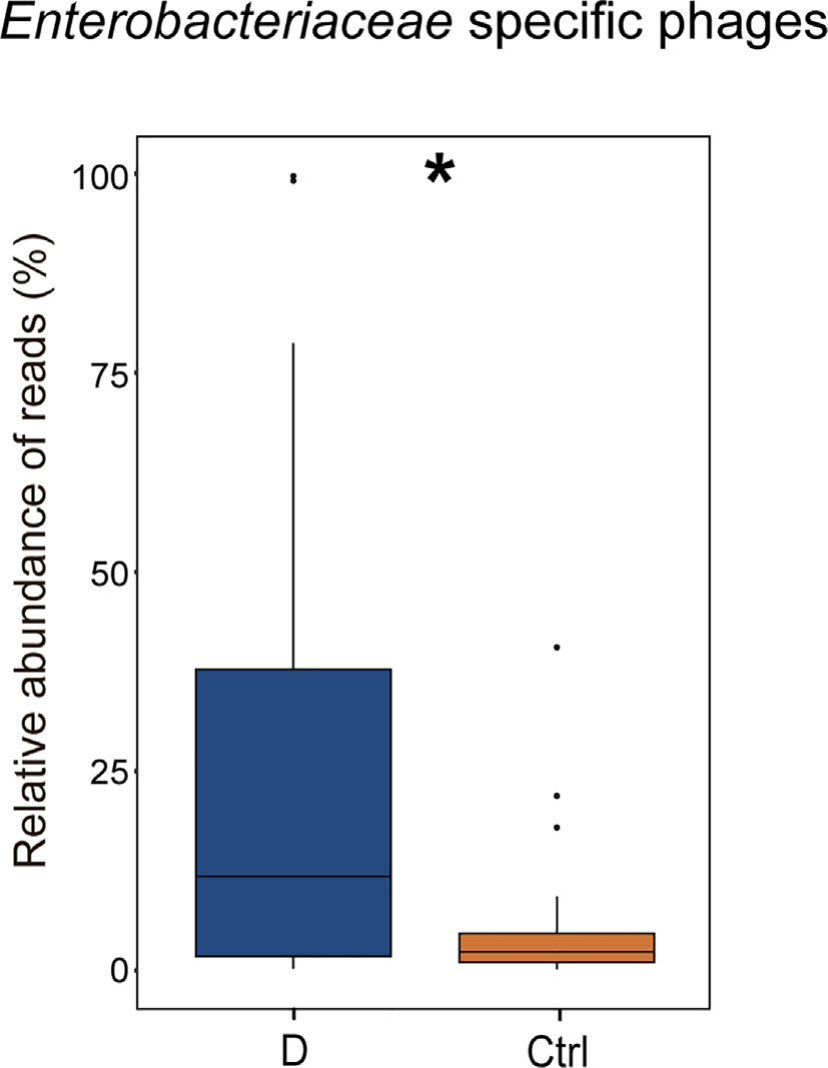
Changes in the relative abundance of *Enterobacteriaceae-*specific phages in T2D group. *p< 0.05.

The results indicated that the relative abundance of gram-negative bacterial phages in the T2D group was slightly higher than that in the Ctrl group (**Supplementary Figure 4A**). The relative abundance of gram-positive bacteriophages changed in the opposite direction. In addition, the most abundant 6 bacterial phyla, *Actinobacteria*, *Bacteroidetes*, *Firmicutes*, *Fusobacteria*, *Proteobacteria*, and *Verrucomicrobia*, were analyzed to explore the changes in gram-positive bacteria and gram-negative bacteria abundances in the T2D group and the Ctrl group, respectively. However, no significant differences in the mentioned bacteria were observed in the T2D group or Ctrl group (**Supplementary Figure 4B**). *Enterobacteriaceae* are typical gram-negative bacteria, and their abundance increased in the T2D group. The alteration in the relative abundance of *Enterobacteriaceae*-specific phages was further evaluated and showed a statistically significant increase in the T2D group (**Figure 7**). The abundance of *Enterobacteriaceae* and the specific phages changed in the same trends.

### Correlations Between the Enteric Phage Community and T2D Disease

**Figure 8:**
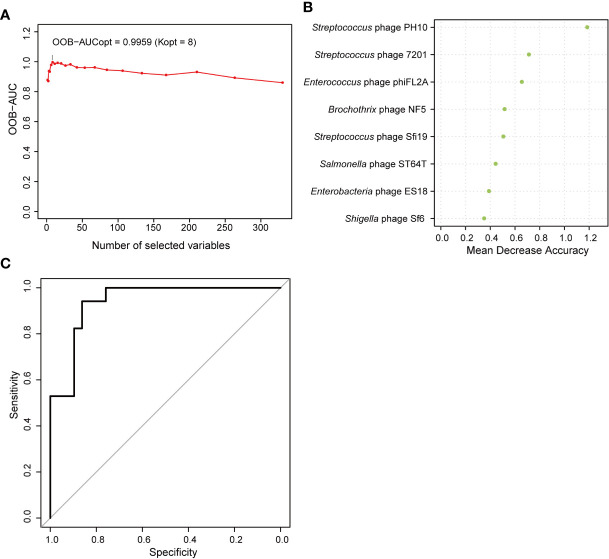
Random forest features for distinguishing type 2 diabetes (T2D) from nondiabetic subjects. **(A)** The change in area under the curve (AUC) values when selecting different numbers of phages. **(B)** Order of the importance of each phage in this model. **(C)** Receiver operating characteristic curve (ROC) analysis of sensitivity and specificity of the eight phages for prediction of T2D.

Correlations between the phageome and T2D disease indicators were evaluated. The results comprehensively presented in **Supplementary Table 4** indicated that there were some significant correlations between several specific phage consortia and 6 T2D indexes, which referred to fasting blood glucose (blood glucose0), fasting insulin (insulin0), insulin 0.5 h after a meal (insulin30), insulin 2 h after a meal (insulin120), highly sensitive C reactive protein (CRP), and free thyroxine. Meanwhile, a model of AUC-RF was produced to maximize the area under the curve (AUC) for a random forest (RF) model. Based on the information in **Supplementary Table 1A**, eight phages were selected based on the algorithm to construct the optimal model, for which the AUC was larger than 0.99 (**Figure 8A**), indicating that these phages could distinguish the T2D from nondiabetic subjects with good performance. In this model, three *Streptococcus* phages, three *Enterobacteriaceae*-specific phages, one *Enterococcus* phage, and one *Brochothrix* phage were significantly selected (**Figure 8B**). Then ROC prediction of these eight phages for T2D was displayed in **Figure 8C** for testing the sensitivity and specificity.

## Discussion

As a typical metabolic syndrome disease, T2D is known to involve complex cellular and molecular mechanisms, leading to dysregulated glucose homeostasis in the body, while the microbiota has been proven to be one of the fundamental factors influencing T2D development. Phage communities are an important component of the microbiota, containing both bacteriophages and the genetic elements of phages integrated into the bacterial genome, representing the phage particles and prophages, respectively, with the ability to modify the microbiota or participate in the horizontal transfer of host genes. Regarding the technical routes for investigating phageomes, a metagenome-dependent method and a VLP isolation-dependent method have been used (Reyes et al., 2010; Norman et al., 2015; Rascovan et al., 2016; Shkoporov et al., 2018; Moreno-Gallego et al., 2019). A previous study using metagenomic data analysis investigated the comprehensive DNA phageome containing both the genomes of phage particles and the genetic elements of prophages in patients with T2D for the first time (Ma et al., 2018). In the present study, we used a method based on virus-like particle isolation and focused on extracellular phages, which could directly regulate the microbiota and thus affect the physiology of the human host. Clinical studies have shown that phage filtrate can achieve good clinical results in patients with severe diarrhea caused by *Clostridioides difficile* (Ott et al., 2017). It also confirmed the regulation of extracellular phage on microecological systems.

Viruses are divided into 152 families based on the latest (2019) report by the International Committee for the Taxonomy of Viruses (ICTV, Virus Metadata Repository: version September 9, 2019; MSL34). The ICTV report divided RNA phages into only two families; *Cystoviridae* with the genus *Cystovirus*, and *Leviviridae*, with two genera, *Levivirus* and *Allolevivirus* (Poranen et al., 2017). While the genome of phages can be DNA or RNA, we focused on the abundant DNA phageome in this study. The study above based on metagenomic analysis revealed an increase in the number of gut phages and an elevation in the relative abundance in the T2D group (Ma et al., 2018). Using the VLP isolation-based method in our study, no changes in abundance of the phage families were found between the T2D group and Ctrl group (**Figure 3B**). Regardless of the differences in the structures of the phageome between the two study cohorts, such divergence illustrated that the study results depend on the chosen research methods. Nevertheless, alterations in phage community were discovered when the phages were clustered according to their bacterial hosts (**Figures 3A, C**).

Remarkably, phages in *Klebsiella* bacteria and *Shigella* bacteria, which are the most concerning *Enterobacteriaceae*, increased in the T2D group. Then, the changes in the relative abundance of all the detected *Enterobacteriaceae*-specific phages between the two groups were assessed and showed a statistically significant increase in the T2D group (**Figure 7**). Meanwhile, the bacteria belonging to the family *Enterobacteriaceae* were significantly increased in T2D patients compared with those in nondiabetic controls (**Figure 4D**). The family *Enterobacteriaceae* is a typical cluster of gram-negative bacteria and is known as a bacterial family enriched in opportunistic pathogens, including *Escherichia*, *Shigella*, *Klebsiella* and *Salmonella*. It’s well known that LPS can be released from gram-negative bacteria, leading to systemic subclinical inflammation and affecting insulin sensitivity (Mehta et al., 2010). Therefore, we speculated that the alteration in gram-negative bacteria and their corresponding phages might lead to elevation circulating LPS content.

**Figure 9:**
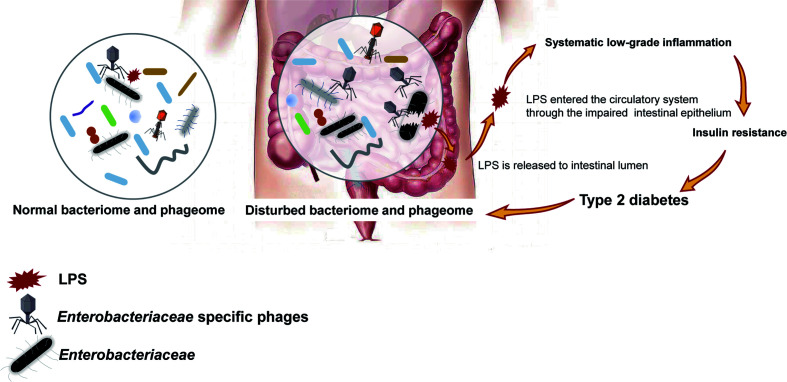
Schematic of the speculated mechanism of how phageome alterations influence the development of type 2 diabetes (T2D). Elevated levels of both *Enterobacteriaceae*-specific phages and members of *Enterobacteriaceae* family were found in the enteric microbiome of T2D patients, which might lead to an increase in circulating lipopolysaccharide (LPS) content and a subsequent systemic inflammation response, resulting in insulin resistance. Arrows present the mechanisms confirmed by previous studies.

To confirm our hypothesis above, the relative abundance of phages whose putative hosts are gram-negative bacteria in the T2D group and Ctrl group were compared, and an increasing trend was found in the T2D group (**Supplementary Figure 4A**). At the same time, the relative abundance of gram-negative bacteria in the T2D group was also evaluated and showed no obvious changes (**Supplementary Figure 4B**). Based on the experimental evidence above, we postulated a pathway *via* which the phage component in the microbiota influences the pathogenesis of T2D (**Figure 9**). That is, an enhanced level of *Enterobacteriaceae* and their specific phages provided a basis for the potential intensified lysis of *Enterobacteriaceae* bacteria under pathological status of T2D, leading to an increase in serum LPS content and then the development or aggravation of T2D. The structures of LPS in different bacteria differ greatly (Navarro et al., 2007), so their pathological consequences also vary. Because *Enterobacteriaceae* have received much attention in traditional infection diseases, the possible pathogenic mechanisms linking *Enterobacteriaceae* family-phages-LPS in T2D should be deeply explored.

Studies have revealed a depletion in universal butyrate-producing bacteria as well as an increase in some opportunistic pathogens in T2D patients (Qin et al., 2012; Jia et al., 2017; Li et al., 2017; Rath et al., 2018). In a metagenomic study performed by a Chinese group, the depleted butyrate-producing bacteria included *Clostridiales* sp. SS3/4, *Eubacterium rectale*, *Faecalibacterium prausnitzii*, *Roseburia intestinalis* and *Roseburia inulinivorans*. The opportunistic pathogens include *Bacteroides caccae*, *Clostridium hathewayi*, *Eggerthella lenta*, and *Escherichia coli* (Qin et al., 2012). From the *16S rRNA* sequencing data processed in this study, the abundance of the butyrate-producing bacteria *Phascolarctobacterium* and *Megasphaera* decreased in T2D patients. *Clostridium* XIVb, which could contribute to the maintenance of gut homeostasis (Lopetuso et al., 2013), was also depleted in the T2D group (**Figure 4E**).

Correlation analysis of the most abundant phages and bacterial genera showed a slightly strengthened correlation in disease status, and most of the correlations were positive correlations (**Figure 5A**), which is in contrast to a previous study about the phages in the gut mucosa in ulcerative colitis (Zuo et al., 2019). However, the mechanism is not clear. The relative abundance of phages and their putative host bacteria in the fecal samples of neither T2D patients nor nondiabetic individuals showed linear correlations (**Figure 5B**). This result could be explained from the perspective of the dynamic relationship of bacteria-phages. Phages not only live in bacteria but also lyse their host bacteria, which have been described as “predator-prey” dynamic models. Additionally, there are also “kill-the-winner” and “arms-race” dynamics at the same time (Koskella and Brockhurst, 2014).

In the last part of our study, we presented numerous phages correlated with T2D disease indicators, including fasting blood glucose, fasting insulin, insulin 0.5 h after a meal, insulin 2 h after a meal, hs-CRP, and free thyroxine (**Supplementary Table 4**). Meanwhile, only a few genera of bacterial hosts have significant correlations with each T2D indicator (**Supplementary Table 5**). Changes in the disease-associated phageome might be more sensitive than those in the bacterial microbiome. Furthermore, the AUC-RF model indicated that eight phages are sufficient for distinguishing T2D from nondiabetic subjects (**Figure 8**).

Generally, OGTT is a routine reliable clinical diagnostic method for T2D, and thus, the detection of specific phages seems unnecessary. However, when from another perspective, since metabolic diseases are usually related to lifestyle habits, such as diet, which could affect the microbiome, the detection of “high-risk” microbiota such as those eight phages in the physical examination project would be reasonable. They may be used as “prediagnosis” biomarkers; upon modulating the microbiome through adjusting lifestyle, the risk of metabolic disease might be reduced. In addition, our data demonstrated the potential of the phageome as a diagnostic indicator of disease. For diseases that lack highly specific diagnostic indicators, the phageome is worthy of consideration as an auxiliary diagnostic indicator.

This study is dependent on the database, not only for mapping viral sequencing, but also for viral taxonomical classification. As far as we know, except for POGs database, some other bioinformatics tools such as VirSorter (Roux et al., 2015) and VirMiner (Zheng et al., 2019) based on databases including RefSeqABVir (Roux et al., 2015), IMG/VR (Paez-Espino et al., 2017), and an updated POG (uPOG) database (Roux et al., 2015), and so on, perform well in viral signals detecting and even functional annotation, phage contig identification and phage-host relationship prediction. Many excellent virome studies using these tools were performed (Shkoporov et al., 2019; Shkoporov and Hill, 2019).

According to previous publications, gut virome yields 75%–99% of reads that couldn’t significantly align to any known viral genome (Aggarwala et al., 2017). Another study revealed that only 1.8% of the assembled putative non-redundant complete and partial viral genomes could be assigned to known viral taxa (Shkoporov et al., 2018). Undeniably, virome analyses nowadays are depended on existing viral genome databases and are limited by them at the same time. For current studies, multiple factors involving diverse informatics pipelines, various databases, different methods for VLPs isolation and viral DNA amplification could result in differential results. In order to make researches in this field more comparable, a “standard operational process” is supposed to be set in virome analysis.

Based on the available data, a comparative analysis of enteric phageome in T2D patients and nondiabetic individuals recruited from Shanghai, China, was conducted in this study. Alterations in bacteriophages predicted to infect *Enterobacteriaceae* in the gut was observed in this study, which was expected to be a source of systemic LPS in T2D patients and was likely to shed considerable light on the overall understanding of the interactions between the microbiome and metabolic diseases. Phages related to T2D were mined, and eight phages performed well in distinguishing T2D, which indicated a promising method for disease diagnosis. Modulation of microbiota and phageome, especially targeted the specific phages, could be a prospective therapeutic method in T2D. However, verifications of the results is lacking, which is a deficiency of this study, and is also a valuable work to do in the next stage. In addition to LPS, phages have proved to induce the release of amyloid from *E.coli* biofilms, which may lead to islet amyloidosis in type 2 diabetes and to islet transplant failure. In this study, we did not focus on the level of amyloid (Abedini and Schmidt, 2013; Tetz et al., 2019). Hypoglycemic medications, especially metformin, have been proved to change the microbiota, and result in the increased abundance of *Escherchia* species (Forslund et al., 2017; Wu et al., 2017). However, we didn’t take into account the effects caused by hypoglycemic medications in this study.

It is urgent that microbiome-related studies should be closely integrated with clinical studies. The indicator of bacterial microbiome changes in disease status has been confirmed to be a valuable auxiliary measure in clinical diagnosis (Koskella and Brockhurst, 2014) and is expected to serve in precision medicine. It is undeniable that with the continuous improvement of phageome research and eventual completion of a standard operating procedure, phageome variation may occupy a place in clinical diagnosis. As a component of the human microbiome that remains to be studied in depth, phageome is thought to be more complicated than the bacterial microbiome. A recent study on a large cohort of populations conducted in China (Yunnan and Hong Kong) has shown that the fecal DNA phage composition can be impacted by both host intrinsic factors (e.g., age, sex, ethnicity, disease status) and extrinsic factors (e.g., geographic residence, diet, stress at work, urbanization-related factors) (Zuo et al., 2020). Additionally, high temporal stability, individual specificity, and correlation with the bacterial microbiome were revealed in a longitudinal metagenomic analysis of fecal viruses in healthy adults in the previous publication (Shkoporov et al., 2019). We found the variation of phgeome in different sample in our study as well, the analysis based on the AUC-RF model found eight phages have the potential biomarker roles, however, due to the limited sample size, what we found is only a preliminary result. Before the phages are considered to be implemented in clinical applications as markers, they must be analyzed in numerous human samples in different regions to ensure the reliability as phage indicators.

## Data Availability Statement

The datasets presented in this study can be found in online repositories. The names of the repository/repositories and accession number(s) can be found in the article/**Supplementary Material**.

## Ethics Statement

The studies involving human participants were reviewed and approved by Shanghai Jiao Tong University Affiliated Sixth People’s Hospital. The patients/participants provided their written informed consent to participate in this study.

## Author Contributions

CLiu, JZ, and XG designed the study. QC performed the “wet experiments” and wrote the manuscript. CLi and QC conducted the analyses. CLiu, JZ, and CLi edited the manuscript. XM, YS, WZ, and YZ recruited the study subjects. XM and JZ provided the clinical information of the patients with T2D. All authors contributed to the article and approved the submitted version.

## Funding 

The authors gratefully acknowledge the financial supported by the Shanghai Municipal Education Commission-Gaofeng Clinical Medicine Grant Support (20161430).

## Conflict of Interest

The authors declare that the research was conducted in the absence of any commercial or financial relationships that could be construed as a potential conflict of interest.
