# Supplementary material for: Enteric Phageome Alterations in Patients With Type 2 Diabetes
Source: Front Cell Infect Microbiol. 2021 Jan 22;10:575084. doi: 10.3389/fcimb.2020.575084 (PMC7862107; doi:10.3389/fcimb.2020.575084)
Supplement: Supplementary file 1 [file DataSheet_1.docx]

Supplementary Material

**Supplementary Table 1. A**. Read counts of the detected 330 species of bacteriophages in each sample.

**B**. The normalized read counts of bacteriophages with significant difference in the T2D group in each sample. The differences in each bacteriophage between the T2D group and the Ctrl group were assessed, and the *p* value and FDR are presented.

**C**. The normalized read counts of bacteriophages classified according to different bacterial hosts in each sample. *p*-values and FDR values associated with differences between the abundance of phages in the T2D group and the Ctrl group are shown in the last two columns.

**D**. The read counts of phages in each sample.

**Supplementary Table 2.** Proportion of various phages at different classification levels corresponding to Fig. S1. Data are shown as percentages.

**Supplementary Table 3.** The OTU table and taxon percent table of the detected bacteria according to *16S rRNA* sequencing.

**Supplementary Table 4.** Phage communities with significant correlations with T2D disease indicators

**Supplementary Table 5.** Bacterial hosts with significant correlations with T2D disease indicators, with a cutoff of an FDR<0.05.

**Supplementary Table 6.** Clinical features and demographic information recorded for each patient with T2D involved in this study.


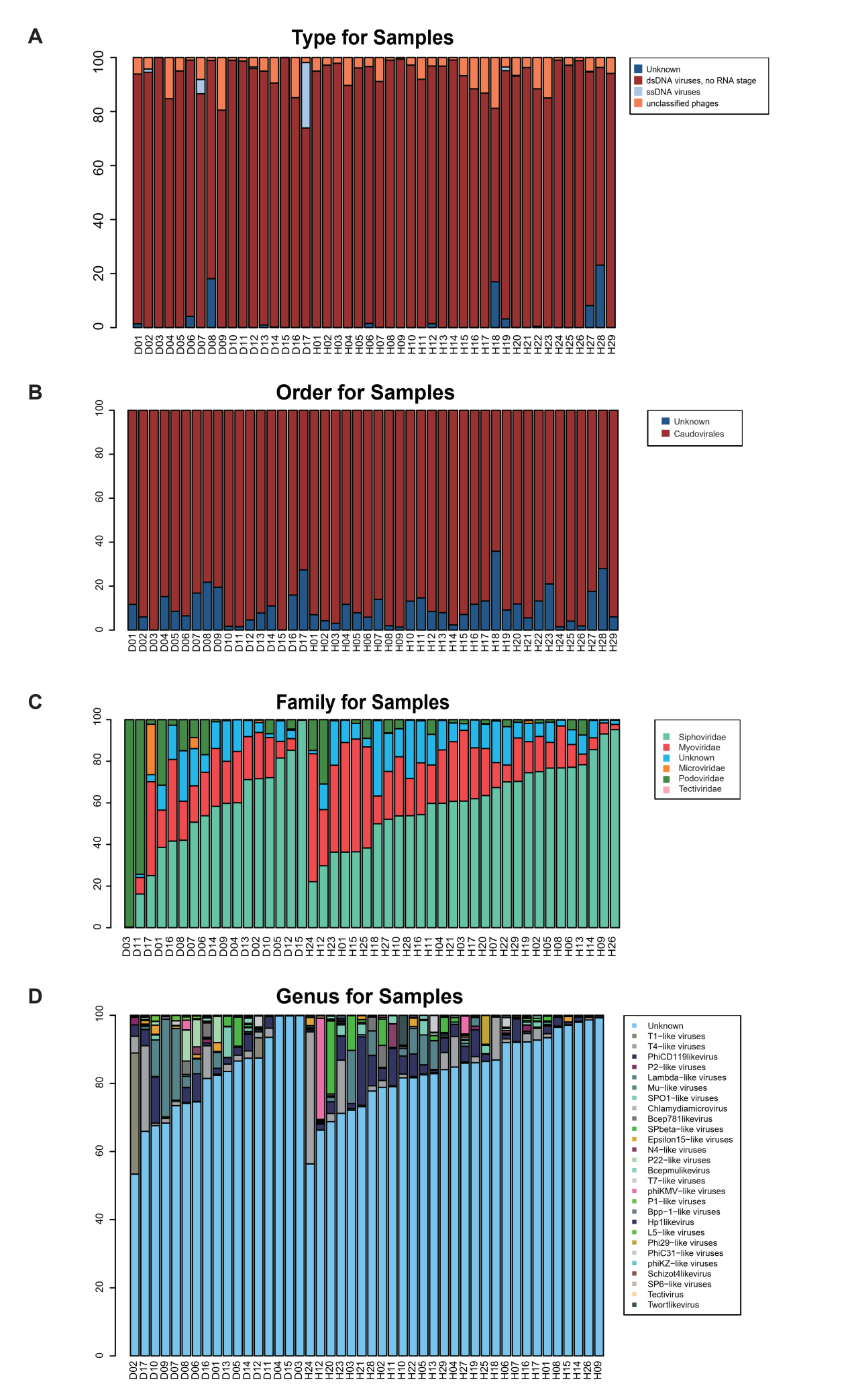


**Supplementary Figure 1.** Phageome features were highly diverse among different individuals. **A** Virus classification derived from sequence alignment. Phage community composition at the order level (**B**), family level (**C**), and genus level (**D**). The samples starting with D and H represent those from T2D patients and nondiabetic individuals, respectively.


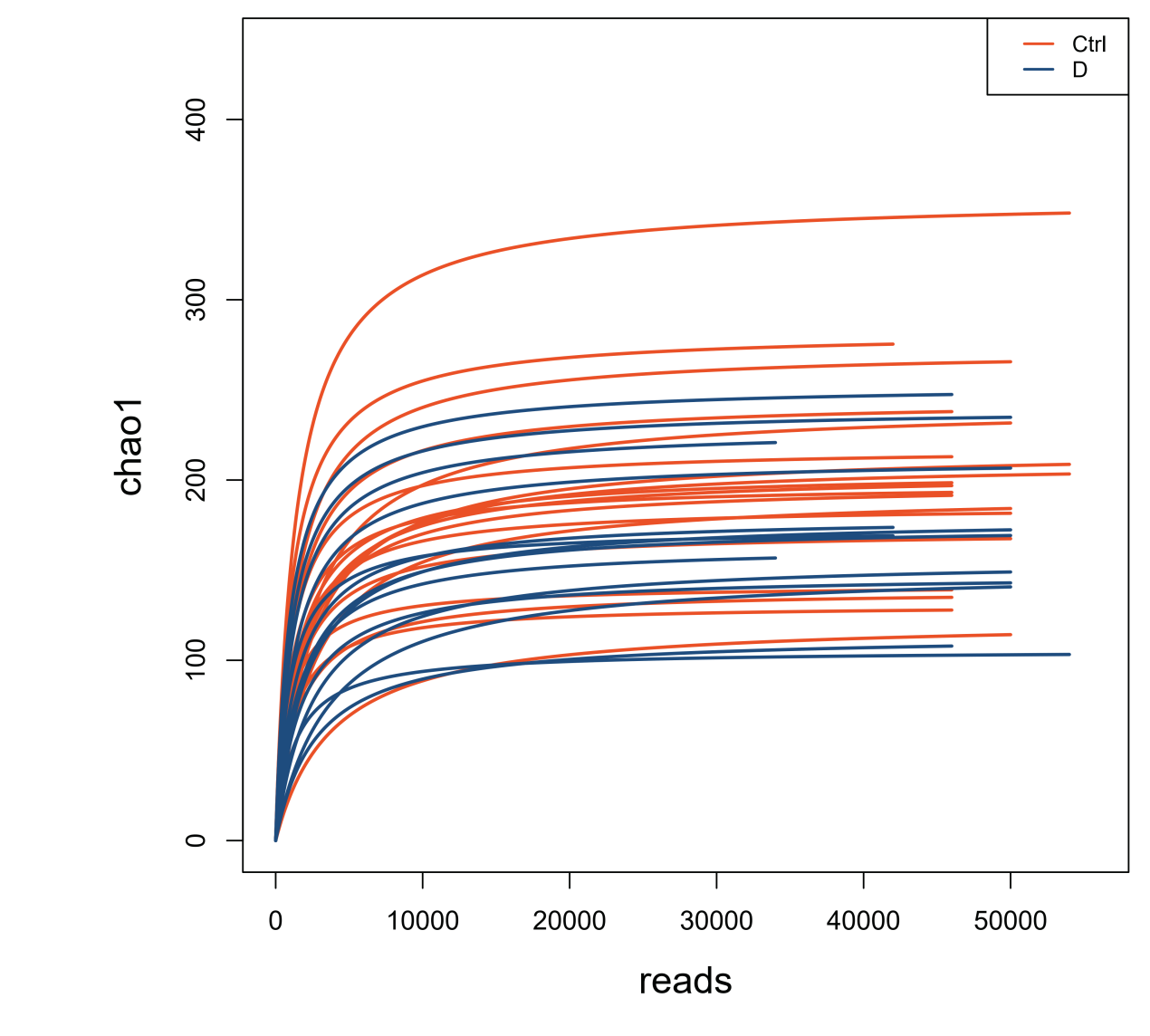


**Supplementary Figure 2.** Rarefaction curves for samples based on Chao1 index.


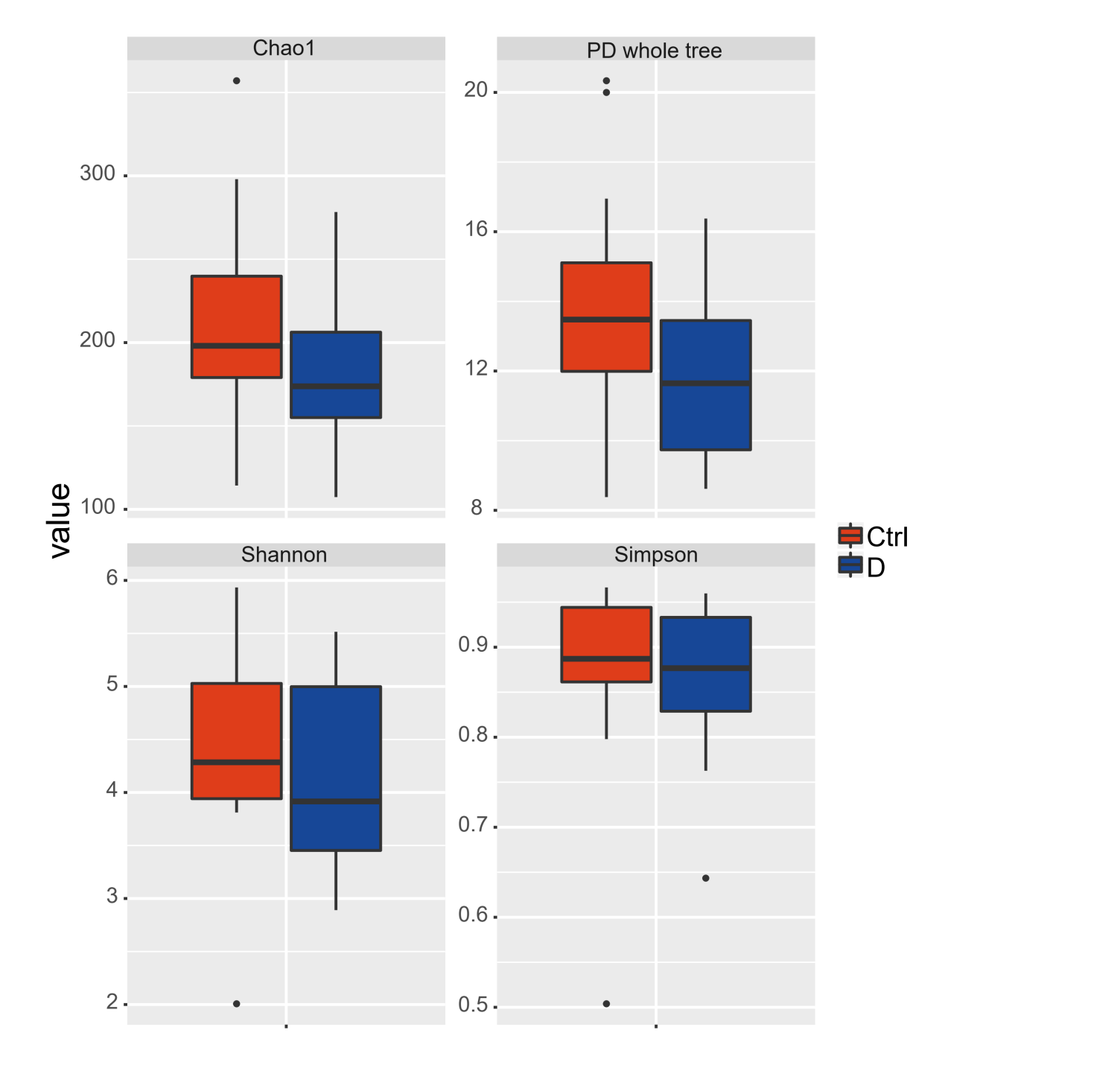


**Supplementary Figure 3.** Box-plots for α-diversity metrics with the indexes Chao1, Shannon, Simpson and PD whole tree.


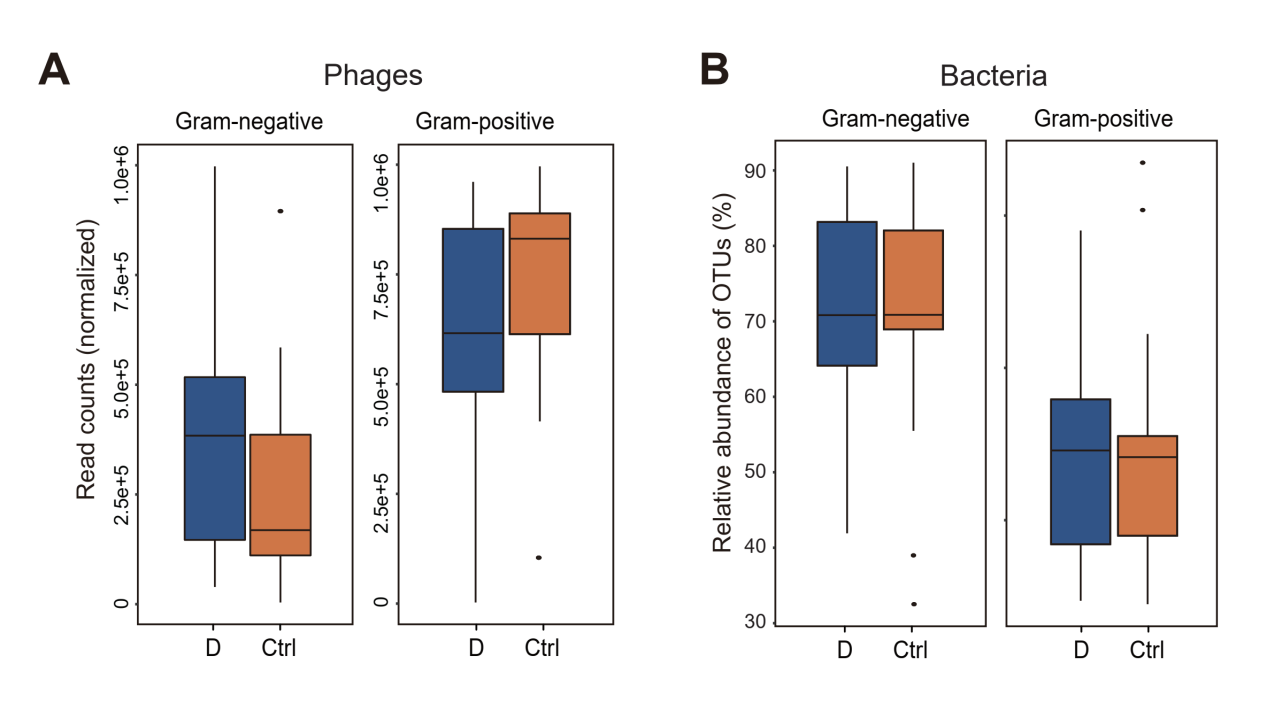


**Supplementary Figure 4.** Changes in the abundance of the gram-positive bacteria, gram-negative bacteria, and their bacteriophages between T2D group and control group. (**A**) Normalized reads number of gram-positive bacterial phages and gram-negative bacterial phages in type 2 diabetes group and nondiabetic control group. (**B**) Changes in the relative abundances of gram-positive bacteria and gram-negative bacteria in both type 2 diabetes group and nondiabetic control group.

| **Supplementary Table 4.** **Phage communities with significant correlation with T2D disease indicators*** | | | | | |
| --- | --- | --- | --- | --- | --- |
|  | **Bacteriophages** |  | **Bacteriophages** |  | **Bacteriophages** |
| InS0 | *Bacillus*_phage_phi29 | InS  30 | *Bacillus*_phage_Fah | InS  120 | *Bacillus*_phage_Fah |
|  | *Bacillus*_phage_Fah |  | *Bacillus*_phage_B103 |  | *Bacillus*_phage_B103 |
|  | *Salmonella*_phage_ST64T |  | *Bacillus*_phage_phi29 |  | *Bacillus*_phage_phi29 |
|  | *Bacillus*_phage_B103 |  | *Salmonella*_phage_ST64T |  | *Salmonella*_phage_ST64T^#^ |
|  | *Lactococcus*_phage_KSY1^#^ |  | *Lactococcus*_phage_KSY1^#^ |  | *Cyanophage*_9515-10a |
|  | *Enterobacteria*_phage_933W_sensu_lato |  | *Cyanophage*_9515-10a |  | *Enterobacteria*_phage_CC31 |
|  | *Lactococcus*_phage_936_sensu_lato |  | *Enterobacteria*_phage_CC31 |  | *Enterococcus*_phage_EF62phi |
|  | *Bordetella*_phage_BPP-1 |  | *Enterococcus*_phage_EF62phi |  | *Klebsiella*_phage_K11^#^ |
|  | *Burkholderia*_phage_phi1026b |  | *Klebsiella*_phage_K11^#^ |  | *Rhodococcus*_phage_ReqiPepy6 |
|  | *Clostridium*_phage_phiCP13O |  | *Rhodococcus*_phage_ReqiPepy6 |  | *Yersinia*_phage_phiR1-37^#^ |
|  | *Enterobacteria*_phage_phiEcoM-GJ1^#^ |  | *Staphylococcus*_phage_187^#^ |  | *Staphylococcus*_phage_phiPVL-CN125 |
|  | *Escherichia*_phage_vB_EcoP_G7C |  | *Staphylococcus*_phage_phiNM3^#^ |  | *Listeria_*phage_B054 |
|  | *Silicibacter*_phage_DSS3phi2 |  | *Yersinia*_phage_phiR1-37^#^ |  | *Staphylococcus*_phage_187^#^ |
|  | *Staphylococcus*_phage_phiETA3^#^ |  | *Myxococcus*_phage_Mx8^#^ |  | *Staphylococcus*_phage_phiNM3^#^ |
|  | *Enterobacteria*_phage_SfV |  | *Staphylococcus*_phage_phiPVL-CN125 |  | *Myxococcus*_phage_Mx8 |
|  | *Mycobacterium*_phage_Trixie^#^ |  | *Enterococcus*_phage_EFAP-1 |  | *Enterococcus*_phage_EFAP-1 |
|  | *Mycobacterium*_phage_244^#^ |  | *Streptococcus*_pyogenes_phage_315.1^#^ |  | *Streptococcus*_phage_O1205 |
|  | *Lactobacillus*_prophage_Lj928 |  | *Klebsiella*_phage_KP15 |  | *Enterococcus*_phage_phiEf11 |
|  | *Streptococcus*_phage_SMP |  | *Thermus*_phage_phiYS40 |  | *Streptococcus*_pyogenes_phage_315.1^#^ |
|  |  |  |  |  | *Thermus*_phage_phiYS40 |
|  |  |  |  |  | *Klebsiella*_phage_KP15 |
|  |  |  |  |  | *Lactococcus*_phage_KSY1^#^ |

InS0, fasting insulin

InS30, 0.5 hr insulin after meal

InS120, 2 hr insulin after meal

| **Supplementary Table 6. Detailed demographic and clinical characteristics of each T2D patient** | | | | | | | | | | |
| --- | --- | --- | --- | --- | --- | --- | --- | --- | --- | --- |
| **subject** | height (cm) | Weight (kg) | systolic pressure (mmHg) | diastolic pressure (mmHg) | HbA_1c_（mmol/mol） | hypersensitive C-reactive protein (mg/l) | triglyceride (mmol/l) | total cholesterol (mmol/l) | LDL-C (mmol/l) | HDL-C (mmol/l) |
| **D01** | 170 | 85 | 180 | 80 | 75.9 (9.1%) | - | 1.18 | 6.76 | 4.94 | 1.1 |
| **D02** | 170 | 59 | 125 | 75 | 104.3 (11.7%) | 0.156 | 0.54 | 3.88 | 2.27 | 1.31 |
| **D03** | 176 | 74 | 130 | 80 | 45.3 (6.3%) | - | 1.24 | 4.38 | 3.16 | 0.92 |
| **D04** | 160 | 56 | 130 | 80 | 51.9 (6.9%) | 0.155 | 1.23 | 4.44 | 2.17 | 1.1 |
| **D05** | 172 | 79 | 118 | 75 | 54.1 (7.1%) | 0.49 | 1.94 | 3.69 | 2.22 | 0.92 |
| **D06** | 158 | 74 | 110 | 70 | 62.8 (7.9%) | 1.13 | 1.73 | 2.57 | 1.17 | 0.91 |
| **D07** | 183 | 70 | 140 | 85 | 97.8 (11.1%) | 0.21 | 0.84 | 4.23 | 2.73 | 1.06 |
| **D08** | 168 | 80 | 130 | 80 | 44.2 (6.2%) | 2.2 | 2.41 | 4.82 | 2.75 | 0.68 |
| **D09** | 176 | 80 | 164 | 74 | 65.0 (8.1%) | 0.87 | 1.67 | 4.14 | 2.63 | 0.86 |
| **D10** | 166 | 76 | 160 | 80 | 62.8 (7.9%) | 0.18 | 1.85 | 3.99 | 2.12 | 1.05 |
| **D11** | 168 | 64 | 112 | 70 | 51.9 (6.9%) | 0.35 | 0.68 | 4.01 | 2.54 | 0.98 |
| **D12** | 177 | 88 | 90 | 70 | 79.2 (9.4%) | 1.36 | 2.78 | 4.7 | 2.77 | 1.05 |
| **D13** | 155 | 70 | 132 | 78 | 56.3 (7.3%) | 4.72 | - | - | - | - |
| **D14** | 163 | 58 | 128 | 84 | 41.0 (5.9%) | 0.51 | 2.81 | 5.6 | 3.49 | 1.33 |
| **D15** | 170 | 65 | 142 | 90 | 65.0 (8.1%) | 0.152 | 0.57 | 2.78 | 1.63 | 1.02 |
| **D16** | 165 | 60 | 130 | 75 | 85.8 (10%) | 0.33 | 0.71 | 5.91 | 4.23 | 1.63 |
| **D17** | 170 | 77 | 130 | 70 | 90.1 (10.4%) | 0.62 | 1 | 3.26 | 1.53 | 1.2 |

LDL-C= low density lipoprotein cholesterol, HDL-C= high density lipoprotein cholesterol

Continued

| **subject** | GPT  (U/l) | GOT  (U/l) | AKP  (U/l) | γ-GT  (U/l) | TBil  (μmol/l) | DBIL  (μmol/l) | TBA  (μmol/l) | GA-L  (g/l) | OCN  (ng/ml) | BUN  (mmol/l) | CRE  (μmol/l) | UA  (μmol/l) | FT3  (pmol/l) | FT  (pmol/l) | TSH  (mIU/l) |
| --- | --- | --- | --- | --- | --- | --- | --- | --- | --- | --- | --- | --- | --- | --- | --- |
| **D01** | 14 | 14 | 91 | 22 | 11.6 | 3.3 | 3.5 | 19.6 | 17.03 | 6.9 | 114 | 343 | 3.77 | 18.65 | 1.25 |
| **D02** | - | 14 | - | - | - | - | - | 33.1 | 22.02 | - | - | - | 3.71 | 17.58 | 1.7 |
| **D03** | 14 | 12 | 58 | 28 | 5.5 | 2.1 | 3.3 | 13.9 | 17.56 | 6.2 | 94 | 410 | 4.62 | 16.85 | 0.92 |
| **D04** | 10 | 13 | 60 | 14 | 10.8 | 3.5 | 4.3 | 16.5 | 17.9 | 4.8 | 44 | 230 | 4.63 | 18.51 | 4.11 |
| **D05** | 30 | 20 | 49 | 48 | 27.1 | 8 | 2.4 | 15.2 | 13.52 | 4 | 60 | 347 | 4.72 | 16.71 | 1.29 |
| **D06** | 12 | 12 | 93 | 11 | 13.7 | 5.3 | 4.1 | 18.9 | 22.36 | 4.8 | 82 | 483 | 4.1 | 14.88 | 2.44 |
| **D07** | 12 | 12 | 60 | 17 | 13.2 | 3.9 | 1.7 | 33.9 | 9.74 | 3.4 | 48 | 357 | 5.28 | 24.39 | 0.75 |
| **D08** | 21 | 12 | 61 | 32 | 12.1 | 4.2 | 6 | 15.7 | 14.46 | 5 | 109 | 457 | 4.55 | 14.63 | 1.26 |
| **D09** | 13 | 14 | 54 | 15 | 20.2 | 5.9 | 4.6 | 21.9 | 10.2 | 5.7 | 93 | 416 | 4.5 | 14.42 | 2.34 |
| **D10** | 36 | 24 | 78 | 26 | 15.1 | 5 | 3.5 | 18.1 | 14.63 | 8.2 | 114 | 483 | 4.28 | 19.81 | 2.15 |
| **D11** | 11 | 9 | 98 | 16 | 16.8 | 5.6 | 2.6 | 21.1 | 12.56 | 5.6 | 83 | 218 | 4.75 | 14.44 | 1.96 |
| **D12** | 18 | 14 | 70 | 30 | 22.4 | 5.3 | 2.1 | 25.6 | 13.23 | 6 | 74 | 392 | 4.07 | 15.62 | 1.25 |
| **D13** | 14 | 11 | 79 | 32 | 3.6 | 1.7 | 2.3 | 13.1 | - | 8.2 | 111 | 95 | - | - | - |
| **D14** | 24 | 21 | 76 | 56 | 14.6 | 3.1 | 6.8 | 16.8 | 12.09 | 7.4 | 138 | 381 | 4.47 | 17.22 | 1.93 |
| **D15** | 10 | 12 | 52 | 12 | 13.1 | 4.8 | 1.2 | 19.4 | 11.71 | 9.1 | 84 | 186 | 5.53 | 16.4 | 3.31 |
| **D16** | 20 | 16 | 61 | 12 | 10.2 | 3 | 4.6 | 34.4 | 14.03 | 5.3 | 39 | 84 | 4.11 | 14.52 | 0.62 |
| **D17** | 16 | 11 | 54 | 16 | 10 | 3.7 | 3 | 18 | - | 4.5 | 58 | 245 | 4.65 | 15.36 | 1.41 |

GPT= glutamic-pyruvic transaminase, GOT= glutamic-oxalacetic transaminase, AKP= Alkaline phosphatase, γ-GT= Glutamyl transpeptidase, TBil= total bilirubin DBIL=direct bilirubin, TBA= total bile acid, GA-L= glycated albumin, OCN= osteocalcin, BUN= urea nitrogen, CRE= creatinine, UA= uric acid, FT3= Free triiodothyronine, FT= free thyroxine, TSH= thyroid stimulating hormone

Continued

| **subject** | Blood glucose (mmol/l) | | |  | Insulin (μU/ml) | | |  | C-peptide (ng/ml) | | | LGFR  (ml/min) | RGFR  (ml/min) | Microalbuminuria (mg/l) | | | | |
| --- | --- | --- | --- | --- | --- | --- | --- | --- | --- | --- | --- | --- | --- | --- | --- | --- | --- | --- |
|  | fasting | 0.5 hr | 2 hr |  | fasting | 0.5 hr | 2 hr |  | fasting | 0.5 hr | 2 hr |  |  | MAU1 (24h) | MAU2 (24h) | MAU3 (24h) | UPR |  |
| **D01** | 5.3700 | 6.5900 | 9.9400 |  | 10.79 | 71.9 | 66.16 |  | 0.52 | 0.91 | 2.11 | 29 | 29.6 | 729.0000 | 637.2000 | 652.0000 | 1.05 |  |
| **D02** | 4.6400 | 11.9600 | 7.0500 |  | 12.4 | 53.53 | 25.42 |  | 0.26 | 0.68 | 0.91 | 39.65 | 50.97 | 7.7490 | 7.9920 | 4.3890 | 0.16 |  |
| **D03** | 5.4900 | 11.0700 | 6.9900 |  | 15.37 | 67.82 | 76.6 |  | 3.26 | 6.72 | 9.81 | 58.2 | 53.8 | 7.1760 | 8.3700 | 7.0300 | 0.09 |  |
| **D04** | 5.8800 | 7.4900 | 12.4800 |  | 1.71 | 9.66 | 33.82 |  | 1.03 | 1.76 | 5.65 | 63.8 | 60.5 | 4.2200 | 4.6200 | 6.6600 | 0.04 |  |
| **D05** | 9.4100 | 12.2000 | 12.8200 |  | 13.06 | 48.32 | 63.62 |  | 2.7 | 4.75 | 8.22 | 75.4 | 61.2 | 20.6830 | 21.6700 | 16.3800 | 0.04 |  |
| **D06** | 8.0400 | 10.9800 | 16.0300 |  | 21.4 | 42.28 | 108 |  | 3.59 | 5.99 | 12.52 | 18.49 | 25.27 | - | - | - | - |  |
| **D07** | 7.9700 | 9.8800 | 8.7900 |  | 12.91 | 31.07 | 36.8 |  | 0.51 | 0.87 | 0.93 | 58.4 | 51.54 | 24.6000 | 25.0800 | - | 0.08 |  |
| **D08** | 5.5000 | 9.8300 | 6.3900 |  | 9.92 | 45.13 | 24.16 |  | 2.56 | 5.08 | 5.28 | - | - | 510.0000 | 538.2000 | 468.0000 | 0.9 |  |
| **D09** | 6.6700 | 9.0100 | 8.7600 |  | 19.33 | 74.17 | 110.9 |  | 2.28 | 2.95 | 2.83 | 36.4 | 36.3 | 17.4720 | 24.6000 | 12.4740 | 0.06 |  |
| **D10** | 5.0300 | 9.9100 | 14.9400 |  | 17.78 | 85.49 | 105 |  | 1.73 | 3.66 | 5.06 | 29.9 | 27.5 | 835.5000 | 330.2000 | 404.3000 | 1.28 |  |
| **D11** | 14.2400 | 13.4900 | 19.8800 |  | 155.7 | 254.2 | 262.9 |  | 0.01 | 0.01 | 0.01 | 72 | 63 | 14.2200 | 12.0400 | 12.0000 | 0.11 |  |
| **D12** | 8.8200 | 10.2600 | 15.2200 |  | 11.47 | 32.88 | 97.26 |  | 1.44 | 1.82 | 5.48 | 51.6 | 47.9 | 7.7760 | 12.8000 | 13.3000 | 0.09 |  |
| **D13** | 8.18 | 9.99 | 9.98 |  | - | - | - |  | - | - | - | 27 | 25.3 | 221.1 | 163.02 | 197.11 |  |  |
| **D14** | 5.5000 | 9.4800 | 11.1200 |  | 8.3 | 51.74 | 113.3 |  | 2.69 | 5.62 | 12.48 | 32.78 | 27.11 | 20.0000 | 18.0200 | 17.5500 | 0.07 |  |
| **D15** | 8.1300 | 7.4000 | 18.8600 |  | 16.02 | 36.55 | 46.71 |  | 0.01 | 0.01 | 0.01 | 55.4 | 65.8 | 38.5500 | 51.2100 | 33.6000 | 0.12 |  |
| **D16** | 6.0600 | 12.4300 | 16.7100 |  | 7.07 | 44.29 | 47.4 |  | 0.41 | 0.94 | 1.57 | 63.6 | 58 | 3.5650 | 5.0700 | 5.3400 | 0.01 |  |
| **D17** | 8.1600 | 11.9900 | 15.0300 |  | 130.4 | 299.1 | 350.2 |  | 0.95 | 1.57 | 1.85 | 32.2 | 45.5 | - | - | - | - |  |

LGFR= left glomerular filtration rate, RGFR= right glomerular filtration rate, MAU= microalbuminuria, UPR= urine protein quantitation

Continued

| **subject** | abdomen ultrasound ^a^ | Electromyography ^b^ | left carotid IMT | right carotid IMT | carotid ultrasound diagnosis ^c^ | supplement for carotid ultrasound diagnosis | lower extremity artery ultrasound diagnosis ^d^ | fundus lesion ^e^ |
| --- | --- | --- | --- | --- | --- | --- | --- | --- |
| **D01** | 1 | 1 | 1 | 1 | 3 | - | 3 | 0 |
| **D02** | 0 | 1 | 1 | 1 | 3 | - | 3 | 0 |
| **D03** | 0 | 0 | 1 | 0.8 | 1 | - | 0 | 0 |
| **D04** | 1 | 0 | 0.9 | 0.9 | 1 | - | 3 | 3 |
| **D05** | 1 | 1 | 0.6 | 0.7 | 3 | Right plaque formation | 0 | 0 |
| **D06** | 1 | 1 | 0.7 | 0.7 | 3 | - | 3 | 0 |
| **D07** | 3 | 2 | 0.7 | 0.6 | 1 | - | 0 | 0 |
| **D08** | - | 0 | - | - | - | - | - | 3 |
| **D09** | 1 | 0 | 0.9 | 0.8 | 3 | - | 0 | 3 |
| **D10** | 1 | 1 | 1 | 1 | 3 | - | 3 | 2 |
| **D11** | 0 | 0 | 0.6 | 0.6 | 1 | - | 0 | 0 |
| **D12** | 1 | 1 | 0.9 | 1.1 | 3 | - | 1 | 0 |
| **D13** | 1 | 1 | 0.7 | 0.6 | 3 | Bilateral arteriosclerosis with right plaque formation | 3 | 0 |
| **D14** | 1 | 0 | 0.7 | 0.7 | 1 | - | - | 0 |
| **D15** | 0 | 1 | 1 | 1 | 3 | - | - | 0 |
| **D16** | 0 | 1 | 0.7 | 0.7 | 3 | Right plaque formation | 0 | 3 |
| **D17** | 0 | 1 | 1 | 1 | 3 | - | 3 | 2 |

IMT, intima-media thickness

1. 0= no fatty liver，1= fatty liver，3= fatty liver trend
2. 0= normal，1= abnormal，2= Peripheral neuropathy
3. 0= normal，1= arteriosclerosis，3= artery atherosclerotic plaque
4. 0= normal，1= arteriosclerosis，3= artery atherosclerotic plaque

0= normal，1= arteriosclerosis， 2= retinopathy，3= mild non-proliferative diabetic retinopathy (NPDR)

Continued

| **subject** | Metformin | Sulfonylurea | TZD | DPP4 inhibitor | Glycosidase inhibitors | SGLT2 inhibitor | GLP-1 receptor agonist | Insulin |
| --- | --- | --- | --- | --- | --- | --- | --- | --- |
| **D01** | 1 | 0 | 0 | 0 | 0 | 0 | 0 | 0 |
| **D02** | 1 | 0 | 0 | 0 | 0 | 1 | 0 | 0 |
| **D03** | 1 | 0 | 0 | 0 | 0 | 0 | 0 | 0 |
| **D04** | 0 | 0 | 0 | 0 | 0 | 0 | 0 | 1 |
| **D05** | 0 | 0 | 1 | 1 | 1 | 0 | 0 | 1 |
| **D06** | 1 | 0 | 0 | 0 | 0 | 0 | 0 | 1 |
| **D07** | 0 | 0 | 0 | 1 | 0 | 0 | 0 | 0 |
| **D08** | 0 | 0 | 0 | 0 | 0 | 0 | 0 | 1 |
| **D09** | 0 | 0 | 0 | 0 | 0 | 0 | 0 | 1 |
| **D10** | 1 | 0 | 1 | 0 | 0 | 0 | 0 | 1 |
| **D11** | 0 | 0 | 0 | 0 | 0 | 0 | 0 | 1 |
| **D12** | 0 | 0 | 0 | 0 | 0 | 0 | 0 | 1 |
| **D13** | 0 | 0 | 0 | 0 | 0 | 0 | 0 | 1 |
| **D14** | 0 | 1 | 0 | 0 | 0 | 1 | 0 | 1 |
| **D15** | 0 | 0 | 0 | 0 | 0 | 1 | 0 | 1 |
| **D16** | 0 | 0 | 0 | 0 | 0 | 0 | 0 | 0 |
| **D17** | 0 | 0 | 0 | 0 | 0 | 1 | 0 | 1 |

DDP4, dipeptidyl peptidase 4; SGLT2, sodium-glucose cotransporter-2; GLP-1, glucagon-like peptide 1

0=no 1=yes

| **subject** | ARB/ACEI | CCB | Beta blocker | Diuretic | Antiplatelet | Statin |  |  |
| --- | --- | --- | --- | --- | --- | --- | --- | --- |
| **D01** | 0 | 0 | 1 | 0 | 0 | 0 |  |  |
| **D02** | 0 | 0 | 0 | 0 | 0 | 1 |  |  |
| **D03** | 0 | 0 | 0 | 0 | 0 | 0 |  |  |
| **D04** | 0 | 0 | 0 | 0 | 1 | 1 |  |  |
| **D05** | 1 | 0 | 0 | 0 | 0 | 1 |  |  |
| **D06** | 1 | 1 | 0 | 0 | 1 | 1 |  |  |
| **D07** | 1 | 0 | 0 | 0 | 0 | 0 |  |  |
| **D08** | 0 | 0 | 0 | 0 | 0 | 0 |  |  |
| **D09** | 0 | 0 | 0 | 0 | 0 | 0 |  |  |
| **D10** | 0 | 0 | 0 | 0 | 0 | 1 |  |  |
| **D11** | 0 | 0 | 0 | 0 | 1 | 0 |  |  |
| **D12** | 0 | 0 | 0 | 0 | 0 | 1 |  |  |
| **D13** | 1 | 1 | 0 | 1 | 0 | 1 |  |  |
| **D14** | 1 | 1 | 0 | 0 | 0 | 1 |  |  |
| **D15** | 1 | 0 | 0 | 1 | 1 | 0 |  |  |
| **D16** | 1 | 0 | 0 | 0 | 0 | 0 |  |  |
| **D17** | 0 | 0 | 0 | 0 | 1 | 1 |  |  |

ARB/ACEI, angiotensin-receptor blockers/angiotensin converting enzyme inhibitors; CCB, calcium channel blocker
